# Improving the care of people with long-term conditions in primary care: protocol for the ENHANCE pilot trial

**DOI:** 10.15256/joc.2015.5.60

**Published:** 2015-12-17

**Authors:** Emma L. Healey, Clare Jinks, Valerie A. Tan, Carolyn A. Chew-Graham, Sarah A. Lawton, Elaine Nicholls, Andrew G. Finney, Mark Porcheret, Vince Cooper, Martyn Lewis, Krysia S. Dziedzic, Simon Wathall, Christian D. Mallen

**Affiliations:** ^1^Research Institute for Primary Care & Health Sciences, Keele University, Keele, Staffordshire, UK; ^2^South Staffordshire and Shropshire Healthcare NHS Foundation Trust, St George’s Hospital, Staffordshire, UK; ^3^School of Nursing and Midwifery, Keele University, Clinical Education Centre, University Hospitals of North Midlands NHS Trust, Royal Stoke University Hospital, Stoke-on-Trent, Staffordshire, UK

**Keywords:** Osteoarthritis, depression, anxiety, multimorbidity, primary care, stepped-wedge design, case-finding, integrated care, long-term conditions

## Abstract

**Background:**

Long-term conditions (LTCs) are important determinants of quality of life and healthcare expenditure worldwide. Whilst multimorbidity is increasingly the norm in primary care, clinical guidelines and the delivery of care remain focused on single diseases, resulting in poorer clinical outcomes. Osteoarthritis, and anxiety and/or depression frequently co-occur with other LTCs, yet are seldom prioritized by the patient or clinician, resulting in higher levels of disability, poorer prognosis, and increased healthcare costs.

**Objective:**

To examine the feasibility and acceptability of an integrated approach to LTC management, tackling the under-diagnosis and under-management of osteoarthritis-related pain and anxiety and/or depression in older adults with other LTCs in primary care.

**Design:**

The ENHANCE study is a pilot stepped-wedge cluster randomized controlled trial to test the feasibility and acceptability of a nurse-led ENAHNCE LTC review consultation for identifying, assessing, and managing joint pain, and anxiety and/or depression in patients attending LTC reviews. Specific objectives (process evaluation and research outcomes) will be achieved through a theoretically informed mixed-methods approach using participant self-reported questionnaires, a medical record review, an ENHANCE EMIS template, qualitative interviews, and audio recordings of the ENHANCE LTC review.

**Discussion:**

Success of the pilot trial will be measured against the level of the primary care team engagement, assessment of training delivery, and degree of patient recruitment and retention. Patient satisfaction and treatment fidelity will also be explored.

ISRCTN registry number: 12154418.

## Background and rationale

Long-term conditions (LTCs) are, with their rising prevalence, increasingly important determinants of quality of life (QoL) and healthcare costs in populations worldwide. Over a quarter of the population of England (15.4 million people) has at least one LTC [1]. Domain 2 of the National Health Service (NHS) outcomes framework has adopted the House of Care as a framework to enhance the QoL for people with LTCs [2], and the UK National Institute for Health and Care Excellence (NICE) publish guidance to help clinicians keep abreast of current recommendations and evidence-based care for specific LTCs and to guide commissioning decisions; however, clinical guidelines usually focus on single conditions in isolation. Other major initiatives to improve quality of care and achieve better health for people with LTCs include the Chronic Care Model [3] and the NHS and Social Care Long-Term Conditions Model [4], both of which highlight key system components that need to be addressed, including delivery-system design, decision support, clinical information systems and self-management support.

Patients with LTCs are predominantly managed in primary care by general practitioners (GPs) and by practice nurses, where they utilize 50% of all appointments [1]. Other practitioners may be involved in wider primary care multidisciplinary teams and services, including physiotherapists, pharmacists, mental health practitioners in primary care mental health teams or Improving Access to Psychological Therapies (IAPT) services [5], and other allied health professionals. Primary care is increasingly seen as the optimal setting to deliver care for people with multiple LTCs because of its generalist approach, accessibility (often the first point of contact), efficiency and ability to identify the health needs of the community, and ensuring that there are services in place to manage those needs [1].

Increasingly, people have multimorbidity, defined as the co-occurrence of two or more LTCs in one person [1]. The number of people with three or more conditions is expected to increase from 1.9 million in 2008 to 2.9 million in 2018 [1]. Patients with several LTCs have poorer QoL and clinical outcomes, longer hospital stays, are more costly to the health service [6], and may experience poorer continuity of care [7], polypharmacy [8], and greater treatment burden [9, 10]. Clinical decision-making is more difficult in patients with multimorbidity because clinicians and patients often struggle to balance the risks and benefits of multiple recommended treatments [11], and because patient preference rightly influences the application of clinical and economic evidence [12]. Whilst robust synthesis of clinical and economic evidence informs guidance for single conditions, combining recommendations for patients with multimorbidity can result in harmful or burdensome treatment regimens [13, 14].

The current focus on single condition management means there are several challenges to identifying which groups of patients could receive multimorbidity interventions. The challenges include working out *who* should identify patients to target, *which combinations* of conditions to target, and at *what level of severity* [15, 16]. Smith *et al.* outline that one approach to this could be to take into account those who may not be in need of intervention and target those at higher risk of adverse outcomes (and most likely to benefit) [16].

Musculoskeletal conditions and mental health problems are common and frequently co-exist, both with each other and with other LTCs. Such conditions tend to be under-diagnosed, with priority given by patients and clinicians to the other LTCs that are perceived to be more important (e.g. diabetes) [17, 18]. Osteoarthritis (OA) is the most common musculoskeletal condition in older adults and is one of the diseases with the highest prevalence of comorbidity with other chronic conditions (such as hypertension, cardiovascular diseases, obesity, respiratory diseases, and diabetes) [19]. People with OA are twice as likely to suffer from heart disease and premature mortality as those in the general population [20]. This co-existence has implications for health, well-being, and the use of healthcare resources, as it is estimated that 8.75 million people in the UK have sought treatment for OA [21].

Depression is common, can be recurrent and can affect anyone, with a prevalence in older people thought to be as high as 30% [22]. More than 20% of people with an LTC may be depressed [23, 24]. Anxiety is also common in people with LTCs, illustrated by an estimated prevalence of 10–42% in people with chronic obstructive pulmonary disease (COPD) [25] and 45% in those with chronic pain [26].

Musculoskeletal and mental health problems are often not prioritized by patient or clinician, thus cost-effective and clinically beneficial treatments are frequently not offered to those in most need. Coventry *et al*. suggest that depression is frequently normalized in the presence of LTCs [18], obviating rather than facilitating further assessment and management [27]. Furthermore, studies have highlighted that symptoms due to OA are often not addressed or appropriately managed in primary care settings [17, 28].

It has been suggested that future work might focus on how interventions that draw on the principles of the Chronic Care Model, such as collaborative care, could support primary care practitioners to better recognize and manage depression in patients with other LTCs [18]. In addition, primary care consultations need to move away from Quality and Outcomes Framework (QOF)-dominated protocols to offer more holistic care [29]; however, long-standing divisions between mental and physical health may pose particular problems for both clinicians and patients, and for integration into existing practice [30]. Smith *et al.* conducted a systematic review of primary care and community-based interventions for multimorbidity and found the results of the studies included were mixed [31]. They recognized that while improving outcomes is challenging, targeting specific risk factors in comorbid conditions or functional difficulties may be effective [31]. The need to develop and test effective and targeted interventions for such populations was also highlighted [6, 31].

The aim of this study is to conduct a pilot stepped-wedge trial to examine the feasibility and acceptability of an integrated approach to LTC management; tackling the under-diagnosis and under-management of OA-related joint pain (knee, hip, hand, and foot) and anxiety and/or depression in patients aged 45 years and over with other LTCs in primary care (asthma/COPD/hypertension or ischaemic heart disease/diabetes), within what we have called the ENHANCE LTC review. A mixed-methods approach will be used to meet the overall aim and objectives set out for this study. This study will report on both process and research outcomes enabling the acceptability and feasibility of the practice nurse training, fidelity of delivery of the ENHANCE LTC review, and suitability for a larger randomized controlled trial (RCT) using the stepped-wedge design to be fully assessed. The protocol for this study has been reported using the Standard Protocol Items: Recommendations for Interventional Trials (SPIRIT) recommendations [32]. The trial has been developed in accordance with published definitions of pilot and feasibility studies [33] and recommendations for good practice in their design and evaluation [16, 34–38].

## Methods

### Trial design and setting

**Figure 1:**
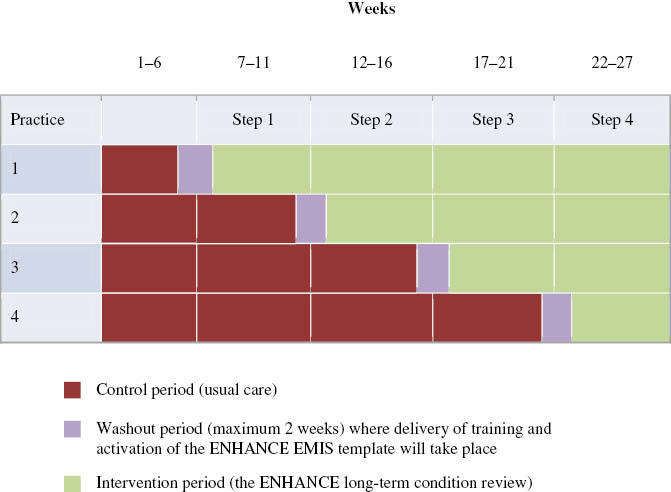
Schematic representation of the stepped-wedge design for the ENHANCE pilot trial.

The ENHANCE trial is a pilot stepped-wedge cluster RCT [39] set in primary care (see Figure 1). The units of randomization are general practices and the units of observation are adults aged 45 years and older consulting for their LTC review at participating general practices.

According to the stepped-wedge design, each general practice recruited to the trial will start by providing ‘usual care’ (the control period), comprising their routine LTC reviews. This will be followed by a washout period (maximum of 2 weeks) where study recruitment will halt to allow the practice nurses to receive training in how to deliver the ENHANCE LTC reviews and to embed the ENHANCE EMIS template within the practice IT system. This will take place at a pre-designated time point, dependent upon the cluster to which the practice has been randomized. The intervention period will then commence during which the ENHANCE LTC review will be embedded within LTC reviews for asthma, COPD, hypertension or ischaemic heart disease, and diabetes, in up to eight consultations per week per practice. It is anticipated that the practices will convert from control to intervention sequentially, every 5 weeks.

In this pilot cluster RCT, GP practices will be eligible to take part if they are: within the geographical areas of NHS Stoke-on-Trent Clinical Commissioning Group (CCG) and NHS North Staffordshire CCG, use the clinical operating system EMIS Web, and have practice nurses who are willing and able to undergo training and participate in both the control and intervention phases.

The balance between scientific considerations and the need for consent is a recognized issue in cluster trials [40]. Following discussion with their practice team, the senior GP partner in each practice will provide informed consent for the practice to participate, acting as ‘guardian’ for patients and their care. GP practice consent to participate will be formalized through written agreements. The care received by eligible patients will be dictated by the period (control or intervention) their practice is currently delivering. The practice nurse will introduce the research study and hand out a study pack to all patients booked into the dedicated consultations who are deemed eligible at the start of the LTC review. The study packs are identical for both control and intervention periods and will include: an invitation letter from the practice to participate in the study; a Participant Information Sheet providing general information on the trial, explaining that delivery of LTC reviews within the practice is being evaluated using patient self-reported outcomes and medical record review; and a self-completion questionnaire, including a consent form and a pre-paid return envelope. A contact number for the study team will be included if the patient wishes to ask any questions before deciding whether or not to participate.

Practices will be asked to invite eight patients per week for the duration of the study. The identification of dedicated ENHANCE consultations will allow practice reception staff to identify and allocate appropriate eligible patients to the consultations and the nurse to focus on handing out the study packs during those consultations only.

The duration of each LTC review varies according to practice and LTC, ranging from 20 to 40 minutes for one condition and up to an hour for those with several conditions. During both the control and intervention periods, practices will be allocated funding to permit five minutes to be added to each ENHANCE LTC review. This is to enable the practice nurse to introduce the study to patients, hand out the study pack and complete the ENHANCE EMIS template (intervention period only). Additional funding will also allow a further 10 minutes to be added twice a week for the practice nurse to generate a list of eligible participants to whom study packs have been provided. This list will then be emailed to a National Institute for Health Research (NIHR) Clinical Research Network (CRN) administrator. An additional 15 minutes will be added to the consultation during the intervention phase, as outlined below.

### Usual care (control)

An initial scoping exercise of the practices within the NHS Stoke-on-Trent CCG and the NHS North Staffordshire CCG revealed variation in ‘usual care’ between practices. Whilst some practices deliver their LTC reviews within dedicated clinics, others provide a more ad hoc appointment system. Whilst we will not ask practices to alter the content of their usual LTC reviews during the control period, we will be asking that patients included in the study are only booked into dedicated study consultations.

### The ENHANCE LTC review (intervention)

The ENHANCE LTC review has been co-designed by researchers, patients, clinicians, and other stakeholders using an implementation of change model and community of practice approach. The development work has been published elsewhere [41]. During the intervention period, practice nurses who have received the study training will deliver the ENHANCE LTC review during the dedicated study consultations, and be supported by the specifically designed study ENHANCE EMIS template.

**Figure 2:**
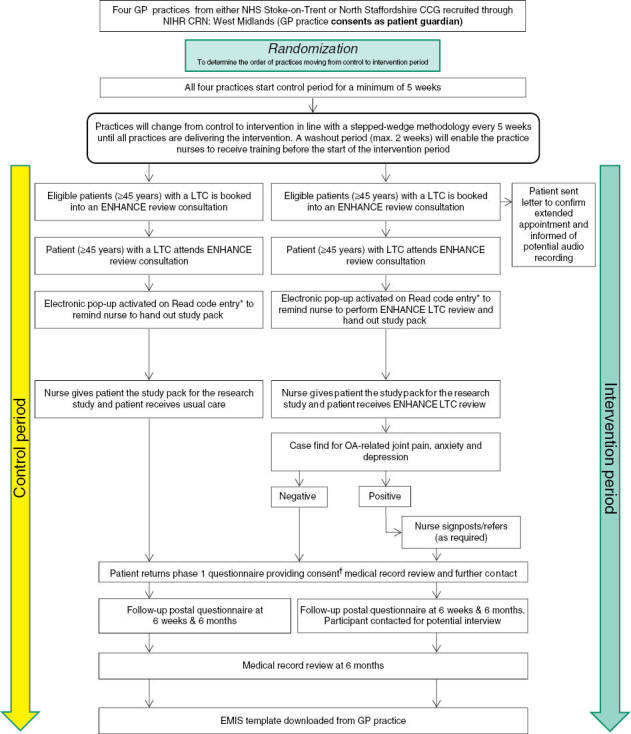
Flow chart demonstrating recruitment of participants into the ENHANCE trial during the control and intervention periods. Long-term conditions (LTC) include asthma, chronic obstructive pulmonary disease, hypertension/ischaemic heart disease, and diabetes. ^*^Based on pre-determined Read codes. ^†^Where consent is incomplete; only phase 1 data will be utilized / medical record review will not be sought / no further contact will be made. CCG, Clinical Commissioning Group; GP, general practitioner; NIHR CRN, National Institute for Health Research Clinical Research Network; NHS, National Health Service; OA, osteoarthritis.

The core components of the ENHANCE LTC review are:

- LTC review consultations set up specifically for the research study
- Usual LTC reviews extended by 15 minutes to integrate:
  - Case-finding for anxiety (Generalized Anxiety Disorder 2-item [GAD-2] questionnaire) [42]/depression (two-question Patient Health Questionnaire [PHQ-2]) [43]/OA-related joint pain (any pain in the hands, hips, knees, or feet)
  - Assessment of anxiety (Generalized Anxiety Disorder 7-item [GAD-7] questionnaire [44]/depression (9-question Patient Health Questionnaire [PHQ-9]) [45]/OA-related joint pain (four questions guided by NICE 2014 working diagnosis of OA [46] (see Figure 2)
  - Negotiation of a management plan which might include facilitating self-management support or signposting/referral to services within or outside the practice
- An ENHANCE LTC review summary card for patients
- An ENHANCE EMIS template to support the review
  - A modified LTC review template, specifically developed for the study, will be embedded within the EMIS system at practice level. The practice nurse will access this template, allowing key information to be recorded and the fidelity of the training and content of the ENHANCE LTC review to be assessed.

### Randomization and allocation concealment

As per stepped-wedge design, all practices will deliver the trial intervention, but they will be randomized to one of four different start dates (approximately 5 weeks apart). Randomization of the practices will be conducted by an independent statistician who will sample practices without replacement using a random generator in Excel. Each practice will act as its own control by delivering usual care for a given period prior to their start date of the intervention.

Allocation concealment for the participating general practices is not possible, and therefore, to minimize recruitment bias, those delivering the intervention (practice nurses) will not be involved in allocating patients to the trial consultations. Eligible participants due for their LTC review will be identified by practice administration staff and allocated an available study appointment.

### Trial population

Our trial population consists of adults attending routine LTC reviews with concordant conditions, i.e. conditions that have shared risk factors. Following randomization, practice administrators will invite approximately 12 patients per week, aged 45 years and over, who are due for their LTC review, to study consultations set up for the duration of the pilot trial (6 months). Patients will be invited to study consultations regardless of previous medical history of OA, anxiety, and depression. ENHANCE appointment slots will be scheduled by practice administrators and eligible patients will be allocated an ENHANCE appointment.

### Eligibility

#### Inclusion criteria

- Registered with the participating GP practice during the specified trial period for that practice
- Aged 45 years and over
- Patients due for their LTC review for asthma/COPD/hypertension or ischaemic heart disease/diabetes.

#### Exclusion criteria

- Vulnerable patients [e.g. patients on the practice register for severe enduring mental ill health (such as unstable schizophrenia/bipolar disorder), significant cognitive impairment (such as dementia), and/or terminal illness]
- Patients who reside in a nursing home and/or have alternative arrangements for LTC care
- Patients unable to read and speak English in order to give informed consent.

### Participant recruitment

All eligible patients booked into the study consultations will be given a study pack at the beginning of their review inviting them to participate in the research. Participants will not be individually randomized; participants recruited during the control and intervention periods of the trial will be asked to take part in a study investigating delivery of LTC reviews in primary care, consisting of the completion of three self-report questionnaires (over a period of 6 months). Participants will also be asked to provide informed consent to follow-up questionnaires, general practice medical record review for the duration of the trial follow-up and further contact from the study team about related studies.

The ENHANCE EMIS template can only be activated and completed once to ensure that patients attend only one study consultation for one LTC, hence are only invited to participate in the study once, thereby avoiding duplicate entries. A list of eligible participants to whom study packs have been provided will be generated by the practice nurse at the end of each clinic and emailed to an NIHR CRN administrator, using a secure ‘nhs.net’ email account. This will allow for the processing of reminders. The GP practices involved in this study have formal agreements with the NIHR CRN West Midlands, whereby staff in the NIHR CRN West Midlands are contracted to work for the practice, to undertake data quality and training functions associated with the GP’s use of their computerized clinical systems, and to undertake administration tasks and functions associated with identifying patients to take part in the research.

Eligible participants will be invited to read the Participant Information Sheet, complete the questionnaire and consent form, and post them back to the research centre using a pre-paid envelope. The Participant Information Sheet will provide a contact number for the research trial coordinator, should the patient wish to ask any questions about the study or clarify the research process before deciding whether or not to participate. The same procedure will be followed for both the intervention and control periods. Similar methods of recruitment have been used successfully in previous studies [e.g. the Primary Care Osteoarthritis Screening Trial (POST) trial ISRCTN: 40721988; the Study of Work and Pain (SWAP) trial ISRCTN: 52269669].

The flow chart summarizes the participant recruitment in both the control and intervention periods (see Figure 2).

### Sample size

We plan to recruit four practices, a number sufficient to test the practical organization of running a stepped-wedge trial. As this is a pilot trial, a formal sample size calculation is not required. However, the number of participants we aim to include in the pilot are detailed below.

To achieve a desired final sample size of approximately 300, we estimate that 800 patients will need to be invited between the four practices over the 6-month trial period, allowing for approximately 50% (*n*=400) consenting to participate, of which 75% will provide 6-month follow-up data. These assumptions are based on trials with similar recruitment methodology conducted at our research centre that were previously mentioned; however, this requires testing in this pilot trial.

### Outcomes

A theoretically informed mixed-methods approach will be employed to assess both feasibility and research method-based outcomes. Data collection methods include participant self-reported questionnaires, a general practice medical record review, an ENHANCE EMIS template, qualitative interviews and audio recordings of the ENHANCE LTC review.

This approach will enable insights into the context-specific processes that influence the application and outcomes of the ENHANCE review [34, 35]. However, a mixed-methods approach can be problematic; therefore, an appropriate theoretical framework is required to facilitate the integration of bodies of evidence, which have distinct philosophical underpinnings [47–49]. A critical realist approach provides a platform from which not only to recognize and measure the effects of interventions using quantitative methods (such as trial methodology or questionnaires) but also to qualitatively investigate social realities, such as organizations, social structures, the actions of agents, and other mechanisms which can influence the implementation or outcomes of interventions [35, 50, 51].

A process evaluation will run in parallel to the trial and aims to answer the following questions:

1. What components of the ENHANCE LTC review were delivered?
2. How was the ENHANCE LTC review delivered?
3. How much was delivered and what happened as a result?

This approach will provide insight into both the implementation and impact of the ENHANCE review on patients with LTCs and members of the practice nursing team (hereafter referred to as ‘practice nurses’). The process evaluation objectives are to assess:

- Feasibility and acceptability of the ENHANCE LTC review, with a particular focus on (i) practice nurse experiences of delivering the ENHANCE LTC review and completing the ENHANCE EMIS template; and (ii) participant experiences of receiving the ENHANCE LTC review, including experiences of treatment burden
- Fidelity of the practice nurse training delivered
- Engagement of general practices participating in the pilot trial
- Engagement of participants in the pilot trial and through follow-up
- Evidence of selection bias in the control and intervention phases
- Recruitment rates in both control and intervention periods
- Follow-up rates at each time point across both control and intervention periods.

We will also undertake a research evaluation to explore factors that might allow the intervention to be implemented and tested appropriately in a larger RCT by exploring the use of specific outcome measures and estimating the parameters needed for a realistic sample size calculation.

The research evaluation objectives are to:

- Examine completion rates of the self-reported outcome measures (for primary and secondary outcome measures)
- Examine completion rates of the ENHANCE EMIS template
- Estimate the parameters needed for a realistic sample size calculation for a larger stepped-wedge cluster RCT
- Examine the feasibility of using a stepped-wedge design within primary care
- Provide an indication of the healthcare and societal costs of implementing the new intervention in comparison with the control group, thereby allowing assessment of potential cost-consequences.

### Quantitative data collection

Self-reported questionnaires, medical record review, and completion of the ENHANCE EMIS template will form the basis of the quantitative data collection.

### Self-reported questionnaires

Participants in the control and intervention periods will be asked to complete self-report questionnaires at three phases: immediately after the LTC review (phase 1), at 6-week (phase 2), and 6-month (phase 3) follow-up. Participants will return their questionnaire to the research centre in a pre-paid envelope. To maximize response rates to the self-report questionnaires, non-responders at phase 1 will be sent a reminder postcard at 1 week and a reminder study pack (invitation letter, questionnaire and Participant Information Sheet with pre-paid envelope) at 2 weeks. Non-responders at phase 2 and 3 will be sent a reminder postcard at 2 weeks and a reminder study pack (invitation letter and questionnaire with pre-paid envelope) at 4 weeks. A minimal data collection (MDC) ‘short’ questionnaire will be sent at 6 weeks (with invitation letter and a pre-paid envelope) and an MDC telephone call will also be conducted by a research nurse for non-responders at 8 weeks (phase 2 and 3 follow-up).

The phase 1 questionnaire will collect information on participant characteristics (e.g. gender, age, employment status), allowing for assessment of potential selection bias by comparing those recruited during the control and intervention periods in each practice. The phase 1 questionnaire will also provide information on any discussions concerning joint pain and/or mood, which took place during the LTC review. This will allow us to investigate acceptability and feasibility on the review and ask whether they received any advice, written information or onward referrals relating to lifestyle changes, medication, and other services. Completion rates of the validated tools will inform their use in a potential larger RCT. The EuroQol 5-dimension, 5-level (EQ-5D-5L) questionnaire [52, 53] will be examined as the primary outcome measure for a larger RCT. Secondary outcome measures to be examined are listed in Table 1.

The ENHANCE team will assess the success of the pilot trial using the following criteria, and the outcome will be reported to the independent Trial Steering Committee (TSC). Our success criteria are that we can engage GP practices to participate and stay in the trial through follow-up (four practices), successfully deliver the training to at least one practice nurse per practice, and recruit (at least 50% of those invited) and retain (75% of those that consent) sufficient participants to the research evaluation.

Table 1 summarizes the outcome measures and their respective time-points of data collection [44, 45, 52–56].

### Medical record review

General practice medical records of consenting participants will be accessed and securely downloaded to obtain information on symptoms, diagnoses, prescriptions, investigations, and referrals. Medical record review of those who provide consent will be undertaken for the 6 months between the ENHANCE LTC review and follow-up, and will allow the study team to examine what activities took place during the reviews, whether patients were referred to other specialists (e.g. local IAPT services, GP, physiotherapist) and potential treatment burden. The data will also allow the study team to examine whether appropriate signposting was being carried out and whether there were any changes to medication during the 6-month trial follow-up. In addition, data on frequency of consultations, prescription use, sickness certification, and secondary care referrals will be examined in terms of likely service cost (an additional question regarding time off work is included in the questionnaire to allow assessment of financial implications due to work absenteeism); these costs will be considered alongside all clinical and process outcomes to allow an indicated cost–consequence framework to inform the suitability of a main trial.

### Use of the ENHANCE EMIS template

Anonymized data will be collected from medical records to examine the use of the ENHANCE EMIS template. The proportion of participants that (i) were asked the case-finding questions; (ii) reported joint pain, anxiety and/or depression; and (iii) were referred or signposted will be calculated. Baseline scores of pain intensity (numerical rating scale) [54], anxiety (GAD-7) [44] and/or depression (PHQ-9) [45] will be examined. Types of referral and signposting will also be explored.

### Qualitative data collection

A sample of ENHANCE LTC reviews will be audio recorded. These data, along with semi-structured interviews with patient participants, practice nurses, and GPs, will form the basis of the qualitative data collection.

### Audio recording of the consultations

A sample of intervention period ENHANCE LTC review consultations will be digitally audio recorded, allowing fidelity of the training to be assessed. Areas of interest will include which elements of the ENHANCE LTC review the practice nurses used, whether the content of the training was evident in practice nurse behaviour, and whether gaps in the ENHANCE LTC review could be demonstrated. In addition, fidelity checking will enable us to describe how the training intervention mapped onto delivery of the ENHANCE LTC review in actual clinical practice.

We anticipate asking each practice nurse to audio record four or five consultations, 2–4 weeks after completion of their intervention period training. This will generate a minimum of 20 recorded consultations, each approximately 30–45 minutes in duration. A digital audio recorder will be used and switched on by the practice nurse (after checking the patient has consented to their consultation being audio recorded) at the start of the consultation.

For those consultations during which reviews may be audio recorded, an additional 10 minutes will be scheduled pre-consultation to allow a qualitative researcher from the study team to discuss the possibility of audio recording, and to obtain initial written consent, if applicable. The researcher will explain to the patient how the audio recording links with the wider research study and will also provide the patient with a study pack for the trial.

Consenting participants will be given a green postcard confirming initial consent to audio recording. This card will be handed to the practice nurse as an indication that consent has been given, therefore allowing audio recording to proceed. After the consultation, the researcher will again discuss consent with the patient before they leave the practice, and once more by telephone 2 days later (at least 48 hours later) to confirm the patient still agrees to give consent for the recording to be kept and analysed by the study team.

The researcher will also obtain written informed consent from the practice nurse for the recording of up to five ENHANCE LTC reviews. Consent will also be obtained after the audio-recorded consultations and by telephone 2 days later (at least 48 hours later) to confirm the practice nurse still consents to the recordings being kept and analysed by the study team.

### Interviews

Semi-structured interviews will be conducted with a sample of patient participants, practice nurses delivering the ENHANCE review and GPs from each practice involved in the study. Tape-assisted (or ‘stimulated’) recall will be used, where possible, to investigate experiences of delivering and receiving the ENHANCE LTC review from the perspective of the practice nurses and participants, respectively. This method facilitates respondent recall and helps to anchor respondents’ reflections in specific consultations [57].

#### Interviews with patient participants

A sample of patient participants identified during the intervention period will be invited to participate in a semi-structured interview. Approximately 20 participants (data collection will continue until category saturation achieved) will be interviewed ensuring purposive sampling of participants with asthma, COPD, hypertension or ischaemic heart disease, and diabetes, those who responded in the affirmative to the case-finding questions for OA-related joint pain, anxiety and/or depression, and those referred onto other services. Where possible, we will use extracts of the audio-recorded consultation to prompt about specific areas of the consultation relating to the aims of the ENHANCE LTC review and use of the case-finding questions for anxiety/depression or OA. These extracts will have been selected by the study team prior to the interview.

Overall, the interviews will explore acceptability of the ENHANCE review, views on the case-finding questions, initial management of newly recognized conditions (referrals and signposting), integration with their index LTC, including potential treatment burden, and their thoughts on the summary card.

#### Interviews with practice nurses

Semi-structured telephone or face-to-face interviews with the practice nurses will explore how the training developed their knowledge and skills to enable them to deliver the ENHANCE LTC review, whether the training could be improved, and what factors helped or hindered implementation of the ENHANCE review.

All practice nurses trained to deliver the ENHANCE LTC review will be invited to participate. A topic guide will allow an exploration of the acceptability and operationalization of the training and ENHANCE LTC review and EMIS template. In addition to the use of a topic guide, we will use extracts of the audio-recorded consultation to prompt discussion about specific areas of the consultation relating to the aims of the ENHANCE LTC review and use of the case-finding questions for anxiety/depression and OA. These extracts will have been selected by the study team prior to the interview.

#### Interviews with GPs

GPs from each of the four practices will be invited to participate in a semi-structured interview at the end of the recruitment period. Where possible, two GPs from each practice will be interviewed to explore the impact of the ENHANCE LTC review on the work of the practice and the GP.

All interviews and audio recordings will be digitally recorded and the interviews will be professionally transcribed verbatim. These methods will allow investigation of the extent to which the ENHANCE review needs modifying to improve either acceptability to patient participants and healthcare professionals, or relevance to the primary care LTC review and integrated care context of its delivery.

### Development of the practice nurse training and ENHANCE EMIS template

In line with previous research, the development of the practice nurse training and the ENHANCE EMIS template will consist of four components [58, 59]: (i) defining the content, (ii) selecting the behaviour change techniques, (iii) deciding on the style of delivery, and (iv) addressing local practicalities.

The content of the training and the ENHANCE EMIS template will be developed by the study team, and informed by the findings from patient and stakeholder workshops and a practice nurse focus group. The behaviour change techniques to be employed within the training will be mapped against a Theoretical Domains Framework [60] identified during the practice nurse focus group [61–63]. In terms of the style of delivery of the training, the principles of the Adult Learning Theory will be drawn upon as its use is well established in the development of courses that support continued professional development [64]. Cochrane Effective Practice and Organisation of Care Group’s reviews and principles of context-bound communication skills training [65] will also be used to inform the style of delivery.

Finally, the practical implications of delivering the training and embedding the ENHANCE EMIS template within the four practices will be considered. The study team will need to take into account the large number of demands already on the practice and work with them to address and agree practicalities, such as the location, duration, and timing of the training, and when the ENHANCE EMIS template can be put in place. It is anticipated that the training will take place in-practice on a one-to-one or small-group basis over 2 half days and will include mentoring from those with expertise in OA and mental health.

We plan to work with a minimum of eight practice nurses (two per practice). Training will take place during a 2-week washout period immediately following the control period, the duration of which is predetermined by randomization. Nurses from each practice will be trained to deliver the ENHANCE LTC review which will be delivered during the ensuing intervention period, and for the remainder of the trial. It is hoped that by training more than one practice nurse per general practice, recruitment can continue throughout the duration of the trial. The training will be preceded by a 1-hour briefing session, available to all practice staff, which will include an overview of the trial and an update of current NICE guidance for OA and anxiety and depression.

### Adverse events

Serious adverse events (SAEs) include death, hospitalization, significant disability or incapacity, any life-threatening circumstance, or any other medically significant occurrence. All practice staff involved in the trial will report immediately to the Principal Investigator (PI) if any identified SAE is experienced by a trial participant. The PI will assess whether the event was related to or resulted from any of the trial procedures or interventions, according to the process laid out in the Keele CTU Standard Operating Procedures. Any SAE considered to be related to the trial procedures will be reported to the main Research Ethics Committee by the PI within 15 days of their awareness of the event. In addition, all such events will be reported to the trial sponsor, TSC, and Data Monitoring Committee (DMC). This is deemed a low-risk trial, as we are investigating the feasibility of enhancing current LTC reviews by incorporating evidence-based management of OA-related joint pain, anxiety, and depression.

### Analysis

#### Quantitative data

Analysis of the quantitative data will be exploratory and provide further data on the feasibility and acceptability of the ENHANCE LTC review and EMIS template. Specifically, descriptive statistics (numbers and percentages, means and standard deviations, and medians and interquartile range) will be used to describe the content of the EMIS template for those in the intervention period of the trial, and the rates of prescription and referrals over 6 months for those consenting to medical record review. Participant characteristics, self-reported at phase 1, will be compared by treatment period, by GP practice, and by practice nurse to explore the balance of patient characteristics by these stratified variables. The percentage of participants consenting to take part in the study will also be reported for each treatment period, along with follow-up rates for the 6-week and 6-month follow-up questionnaires. We will compare recruitment rates and baseline characteristics of participants to assess for potential selection bias. Follow-up data will also be examined in relation to patient characteristics to allow potential attrition bias to be assessed.

Findings from the pilot study will also inform changes in the trial design, recruitment processes, data collection processes, and outcome measures used, that may be required, should the team move to a larger RCT. Specifically, we will explore the suitability of the trial design by assessing the degree of correlation between the self-reported and consultation-based GAD-7 [44] and PHQ-9 [45] outcome measures. Any constraints imposed by the study design should be evident from this comparison; a high correlation is desirable. Possible constraints will be further explored by examining the average time taken for participants to return the phase 1 questionnaire to see how close this is to the date of the initial GP consultation. We will also report completion rates of the self-reported outcome measures, to identify any that are poorly completed, and adapt the content of the self-reported questionnaire accordingly.

Data from the pilot study will also be used to inform a sample size calculation for the main trial by fitting random effects models for the primary outcome of interest (the EQ-5D-5L [52, 53]) at the 6-week and 6-month time points. Models will include fixed effects for treatment period (control or intervention) and time, with time defined as the step number in the stepped-wedge design, from 0 to 4, along with a random effect to represent the GP practice that the patient belongs to [66]. The impact of adjusting for the baseline measure for the outcome of interest as a fixed effect, and the nurse who delivered the intervention, will also be explored. The treatment effect estimates from these models will not be interpreted; however, these models will be used to explore a potential range of estimates for the intra-class correlation coefficient, i.e. the proportion of the individual variance attributable to cluster membership [67] that is needed for a sample size calculation for a main trial. This estimate will be viewed cautiously, however, given that there are only four practices included in the pilot trial.

We have designed the pilot study to include a washout period between the control and intervention phases of the study, both to ensure the nurse training could be fitted into existing workloads and to minimize any delay in the full treatment effect being realised, i.e. to allow time for the nurse to be sufficiently trained to deliver the treatment intervention consistently over time for every patient. We therefore hypothesize that the within-GP practice mean of the primary outcome measure at the 6-week and the 6-month follow-up will not differ greatly, depending on the time period when the intervention was delivered. This will be tested using descriptive statistics and by exploring the coefficients of the term representing ‘time’ in the random effects models used to gain estimates for a sample size calculation for a main trial.

We will also use data from the pilot study to explore the feasibility of a subgroup analysis for a main trial, with the subgroup defined as those participants with co-existing symptoms of anxiety/depression or pain at phase 1 who could potentially benefit from the intervention. The feasibility of this subgroup analysis will be explored by reporting the percentage of participants in each treatment period with co-existing symptoms of anxiety/depression or pain to explore if it is feasible for treatment effects to be calculated only for those participants with the potential to benefit from the intervention. It is anticipated that the proportion of such participants will be similar between treatment periods; therefore, their contribution to the mean effect in each period will be similar; this will be tested in the pilot study data.

#### Qualitative data

One or more researchers will examine the audio recordings of the consultations and use a previously developed tick box scoresheet to assess whether key components of the ENHANCE consultation were demonstrated by the practice nurse.

A tape-assisted recall (or ‘stimulated recall’) approach [57] will be used in the semi-structured practice nurse and participant interviews, with extracts of the consultations being played to stimulate discussion and explore extracts of interest within the consultation. Transcripts of the practice nurse, patient, and GP interviews will be analysed by members of the study team, adopting a constant comparison approach [68, 69], with conceptual themes generated through initial coding of text segments, followed by re-coding and memo writing. Consensus meetings between the qualitative study team will allow discussion and agreement of overarching thematic interpretations. A framework approach will then be used to facilitate interpretation of the data set [70].

## Discussion

Improving the management of people with LTCs has been a key priority of the UK NHS for more than 2 decades [1], with primary care increasingly seen as the optimal setting to deliver care. Yet whilst living with multiple LTCs is increasingly the norm in patients, guidelines and service delivery typically remain focused on single-disease management. For some LTCs, adherence to clinical guidelines is incentivized through inclusion in the QOF component of the General Practice contract, linking financial reward directly to targeted performance. For some conditions not included in QOF (e.g. OA, chronic pain, anxiety), and/or those conditions with more subjective outcomes (e.g. depression), care frequently remains suboptimal and health needs often go unidentified [71, 72]. As Goodwin *et al*. noted, ‘chronic disease management approaches that identify patients on the severity of a single condition may miss multimorbid patients who stand to benefit greatly from improved co-ordination of care’ [6].

Musculoskeletal conditions and mental health problems are common and frequently co-exist, both with each other and with other LTCs. People with comorbid pain and mental health problems have higher levels of disability, a poorer prognosis, and increased healthcare costs; yet the detection and management of these conditions is challenging and frequently suboptimal. Whilst it is recognized that a shift towards integrated care is essential if we are to ensure a holistic approach addressing both physical and mental health problems [18], there is little direct evidence demonstrating what would constitute high quality care or how this may be implemented [6].

The ENHANCE study will examine the feasibility and acceptability of an integrated approach to LTC management, tackling the under-diagnosis and under-management of OA-related pain and anxiety and/or depression in patients with other LTCs in primary care. Satisfaction of those participants who received the ENHANCE review should be at least as acceptable as that of those who received usual care (by comparing mean scores on the General Practice Assessment questionnaire) [55]. Treatment fidelity will further be explored in terms of evaluation of modalities received. Recruitment uptake, follow-up rates, and characteristics of trial participants should be similar in both treatment periods of the study, thus safeguarding against potential selection bias and ensuring validity of any trial data. We will consider a difference in recruitment or follow-up rate of up to 10% to represent an acceptable level of deviation for the pilot trial.

## Ethics approval

The study has been approved by the Greater Manchester East Research Ethics Committee (REC reference: 15/NW/0335).

## Trial monitoring

The ENHANCE trial will be monitored in line with the protocol and Keele’s Clinical Trials Unit Standard Operating Procedures. An independent TSC will monitor the progress of the trial and the DMC will monitor the safety of participants and data integrity. Monitoring will also be undertaken by the approving Research Ethics Committee in the format of annual progress reports and the NIHR Collaborations for Leadership in Applied Health Research and Care West Midlands in the format of quarterly progress reports.

## Public and patient involvement

The trial design and processes have been informed by user involvement in line with our Centre’s strong commitment to involving the public in research, following INVOLVE’s recommendations. Thirteen members of the public attended our patient advisory group meeting held in June 2014 and two members of the public were involved in each of our three stakeholder workshops (in June, July, and September 2014) at which the research question, aim of the trial and design of the intervention were discussed and agreed. Two individuals from the patient advisory group have agreed to be members of our TSC. The remaining members of the patient advisory group assessed our participant information (letters of invitation, Participant Information Sheets, questionnaires and summary card) and their feedback was incorporated in the final versions. All research users are supported by our Centre’s Public and Patient Involvement Coordinator through regular meetings and an annual conference at Keele, with funding support through the Centre’s Centre of Excellence award from Arthritis Research UK.

## Trial sponsor: Keele University

The sponsor will have no role in the design and analysis of the data.

## Figures and Tables

**Table 1:** Outcome measures and timing of data collection.

Measure	Description	Data collection timing				
		Phase 1	Phase 2	Phase2 MDC	Phase3	Phase 3 MDC
Primary outcome measures						
Health outcome	EQ-5D-5L [52, 53]	✓	✓	✓	✓	✓
Secondary outcome measures						
Severity of depression	PHQ-9 [45]	✓	✓	X	✓	X
Severity of anxiety	GAD-7 [44]	✓	✓	X	✓	X
Pain intensity	Numerical rating scale (0–10) [54]	✓	✓	X	✓	X
‘Bothersomeness’ (of pain)	Single question: 1–5 point scale	✓	✓	X	✓	X
Pain interference	Single question: 1–5 point scale	✓	✓	X	✓	X
Health perceptions	Single question: 1–5 point scale	✓	✓	✓	✓	✓
Satisfaction of LTC review	GPAQ nurse assessment [55]	✓	X	X	X	X
Content of LTC review	Topics covered during the review	✓	X	X	X	X
Healthcare utilization	Healthcare utilization questions	X	X	X	✓	X
Work performance	Time off work	X	✓	X	✓	X
Demographics						
Demographics	Gender, date of birth	✓	✓	✓	✓	✓
Socio-economic status	Recent paid job title	✓	✓	X	✓	X
Employment	Current work situation	✓	✓	X	✓	X
Health literacy	Questions to assess health literacy [56]	✓	X	X	X	X

EQ-5D-5L, EuroQol 5-dimension, 5-level questionnaire; GAD-7, Generalized Anxiety Disorder 7-item questionnaire; GPAQ, General Practice Assessment Questionnaire; LTC long-term condition; MDC, minimum data collection; PHQ-9, 9-question Patient Health Questionnaire.
